# Correlation of the endoscopic findings for small and large bowels in pediatric patients with established Crohn’s disease

**DOI:** 10.3164/jcbn.18-83

**Published:** 2018-11-28

**Authors:** Takeru Okuhira, Atsushi Yoden, Tomoki Aomatsu, Masano Akamatsu, Keisuke Inoue, Emiri Kaji, Kimitaka Takitani, Hiroshi Tamai

**Affiliations:** 1 Department of Pediatrics, Osaka Medical College, 2-7 Daigaku-machi, Takatsuki, Osaka 569-8686, Japan; 2 Department of Pediatrics, Osaka Saiseikai Suita Hospital, 1-2 Kawazono-cho, Suita, Osaka 564-0013, Japan; 3 Department of Pediatrics, Hirakata City Hospital, 2-14-1 Kinyahonmachi, Hirakata, Osaka 573-1013, Japan

**Keywords:** capsule endoscopy, fecal calprotectin, pediatric Crohn’s disease, Lewis score

## Abstract

Small bowel capsule endoscopy can detect subtle mucosal lesions in pediatric patients with Crohn’s disease, and our aim was to evaluate its application in established ileocolonic Crohn’s disease. Colonic inflammation was evaluated with the colonic Simple Endoscopic Score for Crohn’s Disease (SES-CD) (excluding the score of the terminal ileum). Small bowel inflammation was evaluated with the Lewis score and/or Capsule Endoscopy Crohn’s Disease Activity Index (CECDAI). A Lewis score <135 was defined as small bowel inactive. A colonic SES-CD of 0 (colonic inactive group) was observed in 22/42 procedures (52.4%), and active small bowel lesions were observed in 11/22 procedures (50.0%). The Lewis score was lower in the colonic inactive group compared to the colonic active group. Correlations between the colonic SES-CD, the Lewis score and CECDAI were weak. The Lewis score and CECDAI in the colonic inactive group had significant correlation with fecal calprotectin levels. We suggest that Crohn’s disease patients without both colonic active lesions and elevation of fecal calprotectin levels may not need to receive small bowel capsule endoscopy due to low incidence of lesions in small bowel.

## Introduction

Crohn’s disease (CD) is a chronic idiopathic inflammatory bowel disease that can affect any segment of the gastrointestinal tract. Small bowel (SB) lesions occur in approximately 60–90% of pediatric patients with CD, but the presence of SB lesion activity does not often correlate with clinical symptom severity.^(1–4)^ Most patients with CD have SB lesions located in the terminal ileum accessible by an ileocolonoscopy (ICS).^(3,5–7)^ Currently, however, the advent of small bowel capsule endoscopy (SBCE) and cross-sectional imaging [particularly computed tomography enterography (CTE) and magnetic resonance enterography (MRE)] have markedly improved the accuracy of SB lesion and jejunum evaluation. Thus, these current modalities are recommended for the evaluation of SB lesions in CD.^(3,8–11)^

SBCE is a noninvasive procedure that can detect subtle SB mucosal lesions without radiation exposure in adults. In addition, the diagnostic yield of SBCE during the evaluation of established non-stricturing small bowel CD is superior to small-bowel follow-through (SBFT), CTE, and MRE.^(12,13)^ SBCE for patients with established CD has a risk of capsule retention. However, the use of a patency capsule (COVIDIEN Ltd., Dublin, Ileland) in patients at risk of capsule retention has proven useful in reducing the retention risk.^(14–16)^

The risk of capsule retention with a patency capsule in pediatric patients with CD is equivalent to the adult risk.^(14,15)^ SBCE has been recommended for evaluating the activity of SB lesions in pediatric patients with CD because of the safety and non-invasiveness of the procedure.^(8,9)^ The Food & Drug Administration (FDA) recommends SBCE for patients aged older than 2 years and invasive esophagogastroduodenoscopic assist for patients with dysphagia. In addition, since SBCE is expensive in some countries,^(17)^ the European Society of Gastrointestinal Endoscopy (ESGE) recommends careful selection of patients with suspected or established CD for the SBCE procedure.^(9,18)^

The use of SBCE is generally determined by a patient’s medical history and/or biomarker levels of CD.^(9,18)^ However, the validity of this approach is not known, and the appropriate biomarkers of SB lesion activity in established CD have not been determined to date.^(19–25)^ We consider that established ileocolonic CD may be detected by ICS and, if the colonic endoscopic findings correlate with small bowel endoscopic findings, small intestinal endoscopic findings may be predicted. However, there is no previous study that reported a correlation between the endoscopic findings of the small bowel and colon in patients with established ileocolonic CD. The aim of this study is to determine the potential application of SBCE in pediatric patients with established CD.

## Materials and Methods

### Patient demographics and clinical data

We examined all records of SBCE and ICS performed on pediatric patients with established ileocolonic CD (aged <19 years) in Osaka Medical College Hospital from August 2012 to August 2017. The interval period between SBCE and ICS was within one month. The exclusion criteria were as follows: An initial diagnosis of CD and use of nonsteroidal anti-inflammatory drugs (NSAIDs) and/or corticosteroids in the last month, a postoperative status, and SBCE was inadequate to evaluate the entire area of the small intestine. Blood tests, including evaluation of the levels of albumin (Alb), C-reactive protein (CRP), erythrocyte sedimentation rate (ESR), fecal hemoglobin, and fecal calprotectin [determined by colloidal gold aggregation (CGA) assay],^(26)^ were performed within one week after the SBCE procedure. Clinical activity was evaluated by the Pediatric Crohn’s Disease Activity Index (PCDAI).^(27)^ PCDAI is often used for pediatric patients with CD, and a PCDAI <10 is defined as clinical remission. The study protocol was approved by the ethics committee of Osaka Medical College (code no. 2240).

### Small bowel capsule endoscopy

All SBCE procedures were performed using the Pill Cam SB2 plus/SB3 and RAPID software (COVIDIEN). All patients received a patency capsule (COVIDIEN) before SBCE to avoid retention and were instructed to prepare for the SBCE procedure with magnesium citrate or polyethylene glycol 2–8 h before the procedure, along with the prokinetic Domperidone and/or mosapride citrate. After the SBCE procedure, we calculated the Lewis score and Capsule Endoscopy Crohn’s Disease Activity Index (CECDAI).^(28–30)^ The Lewis score is the most widely used scoring system for small intestine capsule endoscopic findings for CD and was calculated based on the assessment of villous edema, ulceration, and stenosis for each small bowel tertile divided into equal thirds based on the transit time of the capsule. Following the method in previous literature,^(27)^ we defined a Lewis score <135 as SB inactive and ≥135 as SB active. CECDAI was also used for the assessment of SB CD activity, and inflammation, extent of disease, and presence of strictures were calculated by summation of the proximal and distal scores divided into equal seconds based on the transit time.^(29,30)^ In CECDAI, aphtha and erosions were counted as a score.

### Ileocolonoscopy

Endoscopic findings of the colon were assessed and scored by the Simple Endoscopic Score for Crohn’s Disease (SES-CD).^(31)^ In brief, the colonic SES-CD was calculated based on the ulcer size, ulcerated surface, affected surface, and stenosis for each bowel segment from the rectum to the terminal ileum. A colonic SES-CD = 0 was defined as “colonic inactive”, and a colonic SES-CD ≥1 was defined as “colonic active”.^(25)^

### Statistical analyses

Descriptive statistics were presented as the mean ± SD for normal distributions. Non-normal distributions were expressed as the median [interquartile range (IQR)]. Group differences were evaluated using the Chi-square test or Fisher’s exact test for qualitative variables. The Mann-Whitney *U* test was used when the data did not follow a normal distribution. A *p*<0.05 was considered statistically significant. Spearman’s rank correlation coefficient was used to assess the correlation between the Lewis score, CECDAI, and colonic SES-CD. Statistical analyses were performed using SPSS ver. 23 (IBM Ltd., Armonk, NY).

## Results

### Clinical and demographic characteristics

A total of 42 procedures were performed, and 22 pediatric patients with ileocolonic CD were identified and included in the study (Table 1). The median age was 15.1 (IQR 12.7–17.4) years. Growth retardation was mild (growth height z-score, mean −0.7 ± 2.6), and the PCDAI was not very high (PCDAI, median 5.0, IQR 0–15.0). 5-Aminosalicylate, thiopurine, and infliximab were administered for 34/42 (81.0%) patients, 22/42 (52.4%) patients, and 10/42 (23.8%) patients, respectively. Neither patency capsule retention nor SBCE retention was found in the current study.

### The proportion of active lesions

**Fig. 1:**
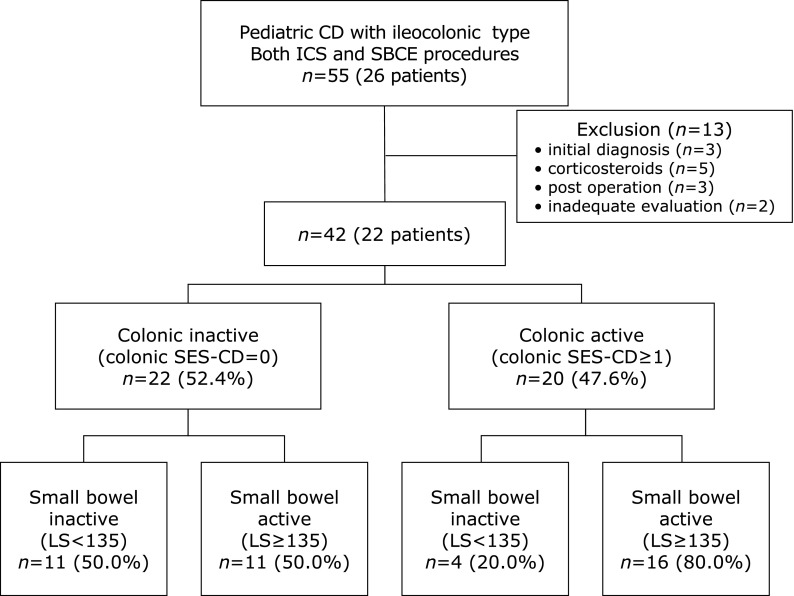
Summary of the small bowel and colonic endoscopic findings. CD, Crohn’s disease; ICS, ileocolonoscopy; SBCE, small bowel capsule endoscopy; SES-CD, Simple Endoscopic Score for Crohn’s Disease; LS, Lewis score.

A total of 22 (52.4%) procedures were performed in the colonic inactive group (colonic SES-CD = 0), and 20 (47.6%) procedures were performed in the colonic active group (colonic SES-CD ≥1) (Fig. 1). In the colonic inactive group, 11/22 procedures (50.0%) were performed on SB active (Lewis score ≥135) patients.

### Frequency differences of SB lesions by type in colonic mucosal activity

**Fig. 2:**
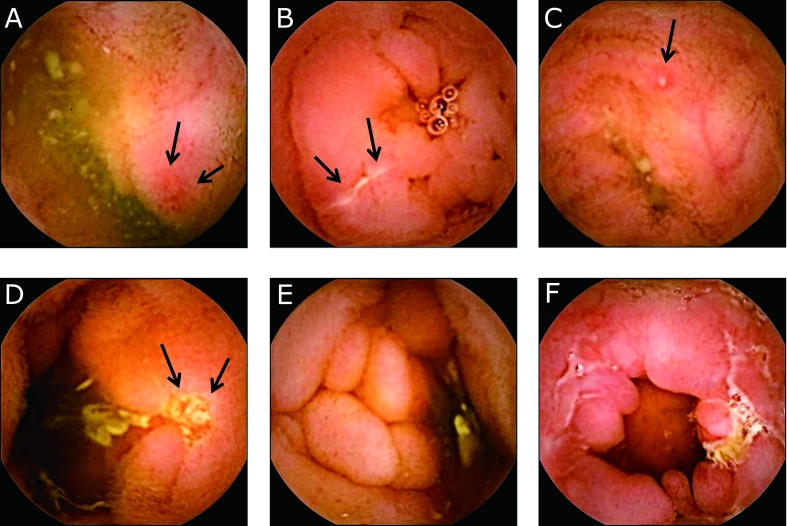
Active lesion of Crohn’s disease in small bowel capsule endoscopy. (A) erythema (*arrow*), (B) erosion (*arrow*), (C) aphtha (*arrow*), (D) ulcer (*arrow*), (E) edematous villi, and (F) stricture.

**Fig. 3:**
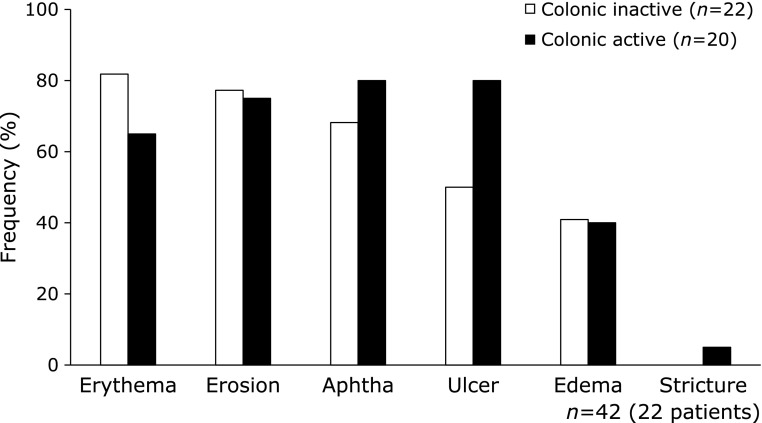
Comparison of active lesion rates in the small bowel between the colonic inactive group and colonic active group. The frequency of active lesions had no statistically significant difference between the colonic inactive group and colonic active group. Statistical analysis was evaluated by Fisher’s exact test.

The frequency of SB lesions by type (Fig. 2) was evaluated between the colonic inactive group and colonic active group (Fig. 3). The frequency of SB lesions with ulcers was higher in the colonic active group compared to the colonic inactive group (*p* = 0.058).

### Comparison of SB mucosal activity and colonic mucosal activity

**Fig. 4:**
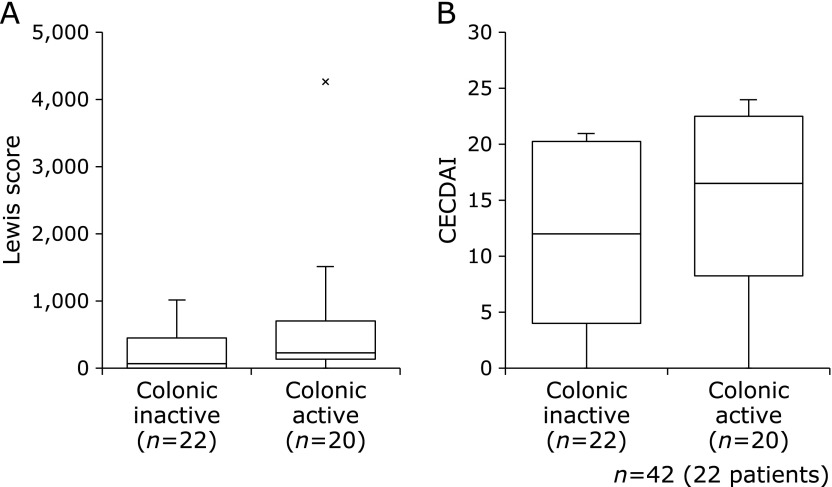
Comparison of small bowel activity between the colonic inactive group and colonic active group. (A) Lewis score and (B) CECDAI. Data were presented as the median. The Lewis score and CECDAI had no statistically significant difference between both groups (Lewis score: *p* = 0.056, CECDAI: *p* = 0.248). Statistical differences were assessed using Mann-Whitney *U* test.

**Fig. 5:**
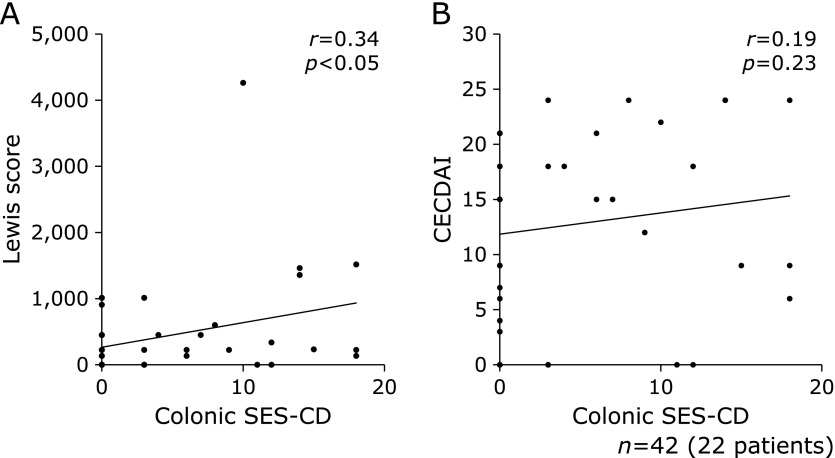
Correlation of small bowel activity with the Lewis score or CECDAI. (A) Lewis score and (B) CECDAI. The correlation between the Lewis score and colonic SES-CD was weak, and CECDAI was not significantly correlated with colonic SES-CD (Lewis score: *r* = 0.337, *p* = 0.029, CECDAI: *r* = 0.190, *p* = 0.229). Statistical differences were assessed using Spearman's rank correlation coefficient.

**Fig. 6:**
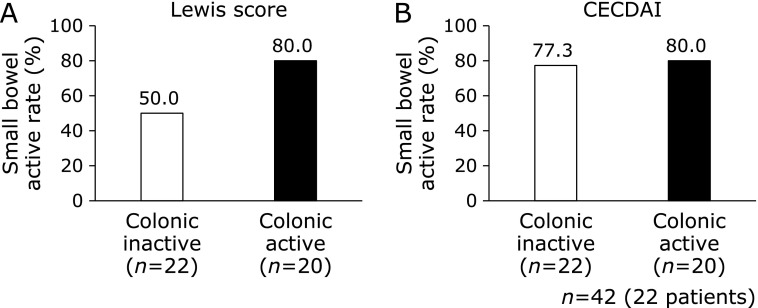
Comparison of small bowel activity between the colonic inactive group and active group. (A) Lewis score and (B) CECDAI. Small bowel inactive was defined as a Lewis score ≥135 or CECDAI ≥3.8. The Lewis score and CECDAI had no statistically significant difference between both groups (Lewis score: *p* = 0.058, CECDAI: *p* = 1.000). Statistical analysis was evaluated by Fisher’s exact test.

The Lewis score in the colonic active group was higher compared to the colonic inactive group (*p* = 0.056), but the CECDAI had no statistically significant difference between both groups (*p* = 0.248) (Fig. 4). Alternatively, there was weak correlation between colonic SES-CD and the Lewis score (Fig. 5a; *r* = 0.34, *p*<0.05), and CECDAI was not significantly correlated with colonic SES-CD (Fig. 5b; *r* = 0.19, *p* = 0.23). There was no statistically significant difference in the frequency of active SB lesions by the Lewis score or CECDAI between both groups (Fig. 6).

### Indicative biomarkers of SB activity

In the colonic inactive group, we compared several factors between the SB inactive and SB active groups. Age and Alb in the SB inactive group were significantly higher compared with those in the SB active group (Table 2; *p* = 0.006, *p* = 0.019, respectively), whereas CRP, ESR, and fecal calprotectin levels in the SB inactive group were lower than those in the SB active group (Table 2; *p* = 0.007, *p* = 0.003, *p* = 0.001, respectively). The proportion of infliximab in the SB inactive group was higher than that in the SB active group (Table 2; *p* = 0.005).

In the colonic inactive group, the Lewis score had a statistically significant correlation with CRP, ESR, and Alb (Table 3A; *r* = 0.551, *p* = 0.008 vs *r* = 0.649, *p* = 0.001 vs *r* = −0.462, *p* = 0.031). Moreover, there was a strong correlation between the Lewis score and fecal calprotectin levels (Table 3A; *r* = 0.827, *p* = 0.00008). The correlation of CECDAI with biomarkers followed a similar pattern to that of the Lewis score (Table 3B).

In the colonic active group, the Lewis score had a statistically significant correlation with CRP, Alb, and fecal hemoglobin level (Table 4A; *r* = 0.634, *p* = 0.003 vs *r* = −0.598, *p* = 0.005 vs *r* = 0.733, *p* = 0.002). However, there was no correlation between the Lewis score and fecal calprotectin levels (Table 4A; *r* = 0.162, *p* = 0.615). The correlation of CECDAI with biomarkers followed a similar pattern to that of the Lewis score (Table 4B).

## Discussion

This is the first study to report the correlation between small bowel and colon endoscopic findings in pediatric ileocolonic CD. Previously, SBFT or ICS was performed to assess SB lesions. However, the SBFT procedure has a lower diagnostic ability than SBCE, and SBCE is particularly superior at the assessment of subtle mucosal inflammation.^(32)^ In recent years, SBCE has been recommended as one of the modalities to assess SB lesions in suspected CD without colonic lesions.^(10)^ In contrast, the usefulness of ICS for the assessment of SB lesions is limited, although subtle lesions may be assessed by it.^(5)^ In the current study, there was no correlation between the mucosal activity of SB and colon in pediatric patients with established ileocolonic CD. Therefore, it may be difficult to evaluate SB activity by ICS alone. However, fecal calprotectin levels were significantly correlated with SB mucosal activity in ileocolonic CD without colonic inflammation. Thus, we conclude that SBCE more effectively assesses the whole small intestine and prevents missing SB ulcers, considering that ICS alone found 50.0% of the SB ulcers in the colonic inactive group.

Colonic mucosal healing in CD has been well discussed;^(33)^ however, there are very few reports on SB mucosal healing in CD.^(19)^ There are some reports on the availability of SBCE;^(12,13)^ however, the prognosis in SB active CD patients must be elucidated. Several studies have established that SBCE findings of SB activity are observed in CD patients even though they have clinical or biomarker remission and that SBCE contributes to determining how patients with CD are managed.^(19,20,34)^ However, determining whether mild SBCE findings affect the management and prognosis of patients with CD remains controversial. We should pay careful attention to the interpretation of SBCE findings, because positive subtle mucosal findings are reported in 80% of the healthy patients.^(35)^ Thus, further investigation will be needed to clarify if finding mild SB lesions in ileocolonic CD contributes to the management strategies or prognosis of patients with CD.

Several studies reported the correlation between SBCE findings and biomarkers including CRP, ESR, and fecal calprotectin levels.^(19–25,36–43)^ The current study indicated that the Lewis score and CECDAI had significant correlations with CRP, ESR, Alb, and fecal calprotectin levels in colonic inactive group. In addition, there was a strong correlation between fecal calprotectin levels and the Lewis score in the colonic inactive group. However, the medians of CRP, ESR, and Alb were almost within normal values, and there was little difference between both groups. It may be difficult to evaluate the results and apply the findings in clinical practice. The fecal calprotectin level differences between the SB inactive and active groups without colonic inflammation were statistically significant, and the fecal calprotectin levels had a strong correlation with the Lewis score and CECDAI. In the current study, there were different strengths of the correlation between fecal calprotectin levels and the scoring systems including the Lewis score and CECDAI (*p* = 0.00008 vs *p* = 0.0002). This difference may be a result of the different scoring mechanisms, because the Lewis score evaluates mainly ulcerated lesions.

The SBCE procedure has some problems including the high cost, burden of the SBCE interpretation, difficulty of cleansing the small intestine, and SBCE swallowing particularly in pediatric patients. The most successful swallowing age for SBCE without using an esophagogastroduodenoscopy is considered to be ≥8 years old,^(44)^ although the FDA approved SBCE for patients ≥2 years old. Modalities other than SBCE, including CTE, MRE, and ultrasound are generally performed for young children.^(45)^ However, it has been reported that SBCE in combination with ICS improves diagnostic accuracy.^(13)^ Based on our findings, fecal calprotectin level may be the most useful biomarker to determine whether SBCE should be performed in pediatric patients with ileocolonic CD. On the other hand, the patients with active colonic lesions are likely to benefit from SBCE, because the Lewis score and the CECDAI in the patients with active colonic lesions were higher than those in the patients without such lesions in the current study. Therefore, both the patients with colonic active lesions and those having no colonic active lesions with elevated calprotectin levels are candidates for SBCE. Alternatively, we consider that it may be unnecessary for CD patients without both colonic active lesions and elevation of calprotectin to receive SBCE due to low incidence of lesions in small bowel.

There are several limitations in the present study. First, the patients’ background of the medication history was different, and any subjects received more than one procedure. This suggests a possibility that the results depend on the type of treatment. Moreover, determining the cut-off value for calprotectin levels were difficult because the number of procedures in the current study was small. Therefore, a larger-scale study will be required to clarify this issue. Second, the present study included less severe cases in the colonic inactive group. There was no significant difference between the SB active and SB inactive without colonic lesion groups regarding PCDAI and growth height z-score. It is contraindicated to perform SBCE for severe stricture CD patients and, therefore, the findings may be biased. We conclude that it is better to perform cross-sectional imaging including CTE and MRE for these severe cases. Third, fecal calprotectin levels in colonic active CD may not be a useful marker for the application of SBCE, because they are affected by colonic active lesions regardless of the presence or absence of SB lesions.

In conclusion, assessment of both colonic mucosal activity by ICS and SB mucosal activity by SBCE in pediatric patients with established ileocolonic CD is important, because there is no correlation between colonic mucosal activity and SB mucosal activity. Therefore, we suggest that CD patients without both colonic active lesions and elevation of calprotectin levels may not need to receive SBCE due to low incidence of lesions in small bowel. Further investigation will be required to clarify this issue.

## Author Contributions

TO, AY and TA designed the study; TO, AY, TA, EK, KI and MA performed procedures; TO collected and analyzed data; TO, AY, TA and KT wrote the manuscript; AY, KT, and TA gave technical support and conceptual advice. HT reviewed and edited the manuscript. All authors read and approved the final manuscript.

## Figures and Tables

**Table 1:** Clinical and demographic characteristics of patients

Procedure		42 (22 patients)
Age (years)	median (IQR)	15.1 (12.7–17.4)
Male/Female		33/9
Height (cm)	mean ± SD	159.3 ± 11.3
Body weight (kg)	mean ± SD	50.5 ± 10.7
Body mass index (kg/m^2^)	mean ± SD	19.7 ± 3.0
Growth height z-score	mean ± SD	–0.7 ± 2.6
PCDAI	median (IQR)	5.0 (0–15.0)
Treatment		
	5-ASA	81.0% (34/42)
	Azathiopurine (or 6-mercaptopurine)	52.4% (22/42)
	Prednisolone	0% (0/42)
	Infliximab	23.8% (10/42)
	Others (no medication or herbal medicine)	14.3% (6/42)

Abbreviations are referred following. PCDAI, Pediatric Crohn’s Disease Activity Index (clinical remission; <10); 5-ASA, 5-aminosalicylic acid.

**Table 2:** Comparison small bowel inactive and active in colonic inactive group

		Small bowel inactive (*n* = 11)	Small bowel active (*n* = 11)	*p* value
Age (years)	median (IQR)	16.3 (15.8–18.3)	12.8 (11.2–15.1)	0.006**
Male/Female		11/0	11/0	0.317
Body mass index (kg/m^2^)	mean ± SD	20.2 ± 2.2	20.8 ± 3.7	0.922
Growth height z-score	mean ± SD	–1.4 ± 2.2	–2.1 ± 2.0	0.577
C-reactive protein (mg/dl)	median (IQR)	0.03 (0.02–0.05)	0.09 (0.07–0.39)	0.007**
ESR (mm/1 h)	median (IQR)	3.0 (2.0–5.0)	8.0 (4.0–11.0)	0.003**
Albumin (g/dl)	mean ± SD	4.7 ± 0.4	4.3 ± 0.3	0.019*
Fecal hemoglobin (ng/ml)	mean ± SD	0.0 ± 0.0	81.6 ± 258.0	0.294
Fecal calprotectin (µg/g)	median (IQR)	98 (47–224)	826 (488–1,235)	0.001**
PCDAI	median (IQR)	5.0 (0.0–5.0)	0 (0–20.0)	0.777
Treatment				
5-ASA		100% (11/11)	72.7% (8/11)	0.069
Azathioprine (or 6-mercaptopurine)		45.5% (5/11)	63.6% (7/11)	0.403
Infliximab		54.5% (6/11)	0% (0/11)	0.005**
Others (no medication or herbal medicine)		0% (0/11)	27.3% (3/11)	0.069

Asterisks represent significant difference and abbreviations are referred following. ESR, erythrocyte sedimentation rate; PCDAI, Pediatric Crohn’s Disease Activity Index (clinical remission; <10); 5-ASA, 5-aminosalicylic acid.

**Table 3:** Correlation between the scores of CD and biomarkers in colonic inactive group

A. Lewis score			
	*n*	*r*	*p*
C-reactive protein (mg/dl)	22	0.551	0.008**
Erythrocyte sedimentation rate (mm/1 h)	22	0.649	0.001**
Albumin (g/dl)	22	–0.462	0.031*
Fecal hemoglobin (ng/ml)	21	0.14	0.545
Fecal calprotectin (µg/g)	16	0.827	0.00008**

B. CECDAI			
	*n*	*r*	*p*
C-reactive protein (mg/dl)	22	0.487	0.022*
Erythrocyte sedimentation rate (mm/1 h)	22	0.453	0.034*
Albumin (g/dl)	22	–0.469	0.028*
Fecal hemoglobin (ng/ml)	21	0.299	0.189
Fecal calprotectin (µg/g)	16	0.796	0.0002**

Asterisks represent significant difference and abbreviations are referred following. CD, Crohn’s Disease; CECDAI, Capsule endoscopy Crohn’s disease activity index.

**Table 4:** Correlation between the scores of CD and biomarkers in colonic active group

A. Lewis score			
	*n*	*r*	*p*
C-reactive protein (mg/dl)	20	0.634	0.003**
Erythrocyte sedimentation rate (mm/1 h)	20	0.347	0.134
Albumin (g/dl)	20	–0.598	0.005**
Fecal hemoglobin (ng/ml)	20	0.733	0.002**
Fecal calprotectin (µg/g)	12	0.162	0.615

B. CECDAI			
	*n*	*r*	*p*
C-reactive protein (mg/dl)	20	0.61	0.004**
Erythrocyte sedimentation rate (mm/1 h)	20	0.173	0.466
Albumin (g/dl)	20	–0.526	0.017*
Fecal hemoglobin (ng/ml)	20	0.494	0.027*
Fecal calprotectin (µg/g)	12	0.139	0.666

Asterisks represent significant difference and abbreviations are referred following. CD, Crohn’s Disease; CECDAI, Capsule endoscopy Crohn’s disease activity index.

## Abbreviations

Alb: albumin
CD: Crohn’s disease
CECDAI: Capsule Endoscopy Crohn’s Disease Activity Index
CGA: colloidal gold aggregation
CRP: C-reactive protein
CTE: computed tomography enterography
ESGE: European Society of Gastrointestinal Endoscopy
ESR: erythrocyte sedimentation rate
FDA: Food & Drug Administration
ICS: ileocolonoscopy
MRE: magnetic resonance enterography
NSAIDs: nonsteroidal anti-inflammatory drugs
PCDAI: Pediatric Crohn’s Disease Activity Index
SB: small bowel
SBCE: small bowel capsule endoscopy
SBFT: small bowel follow-through
SES-CD: Simple Endoscopic Score for Crohn’s Disease
